# Yeast Ste2 receptors as tools for study of mammalian protein kinases and adaptors involved in receptor trafficking

**DOI:** 10.1186/1750-2187-1-2

**Published:** 2006-11-10

**Authors:** Dezhong Yin, Elena Shumay, Hsien-yu Wang, Craig C Malbon

**Affiliations:** 1 Department of Pharmacology, Diabetes & Metabolic Diseases Research Center, School of Medicine, SUNY/Stony Brook, Stony Brook, NY 11794-8651, USA; 2 Physiology & Biophysics, Diabetes & Metabolic Diseases Research Center, School of Medicine, SUNY/Stony Brook, Stony Brook, NY 11794-8651, USA

## Abstract

**Background:**

Mammalian receptors that couple to effectors via heterotrimeric G proteins (*e.g.*, beta _2_-adrenergic receptors) and receptors with intrinsic tyrosine kinase activity (*e.g.*, insulin and IGF-I receptors) constitute the proximal points of two dominant cell signaling pathways. Receptors coupled to G proteins can be substrates for tyrosine kinases, integrating signals from both pathways. Yeast cells, in contrast, display G protein-coupled receptors (*e.g.*, alpha-factor pheromone receptor Ste2) that have evolved in the absence of receptor tyrosine kinases, such as those found in higher organisms. We sought to understand the motifs in G protein-coupled receptors that act as substrates for receptor tyrosine kinases and the functional consequence of such phosphorylation on receptor biology. We expressed in human HEK 293 cells yeast wild-type Ste2 as well as a Ste2 chimera engineered with cytoplasmic domains of the beta_2_-adrenergic receptor and tested receptor sequestration in response to activation of the insulin receptor tyrosine kinase.

**Results:**

The yeast Ste2 was successfully expressed in HEK 293 cells. In response to alpha-factor, Ste2 signals to the mitogen-activated protein kinase pathway and internalizes. Wash out of agonist and addition of antagonist does not lead to Ste2 recycling to the cell membrane. Internalized Ste2 is not significantly degraded. Beta_2_-adrenergic receptors display internalization in response to agonist (isoproterenol), but rapidly recycle to the cell membrane following wash out of agonist and addition of antagonist. Beta_2_-adrenergic receptors display internalization in response to activation of insulin receptors (*i.e.*, cross-regulation), whereas Ste2 does not. Substitution of the cytoplasmic domains of the β_2_-adrenergic receptor for those of Ste2 creates a Ste2/beta_2_-adrenergic receptor chimera displaying insulin-stimulated internalization.

**Conclusion:**

Chimera composed of yeast Ste2 into which domains of mammalian G protein-coupled receptors have been substituted, when expressed in animal cells, provide a unique tool for study of the regulation of G protein-coupled receptor trafficking by mammalian receptor tyrosine kinases and adaptor proteins.

## Background

G protein-coupled receptors (GPCR) are intrinsic membrane proteins that transduce agonist binding into activation of heterotrimeric G proteins, a paradigm for cell signaling extending from yeast to mammals [1]. The yeast STE2 gene product is a heptahelical, GPCR that binds and transduces intracellular signaling from the α-factor pheromone [2,3]. Recently we succeeded in expressing the yeast α-factor pheromone receptor Ste2 in human HEK293 cells, demonstrating that Ste2 was fully functional and capable of stimulating activation of the mitogen-activated protein kinase Erk1/2 in response to α-factor [4]. In yeast, activation of Ste2 leads to the internalization, ubiquitination, and rapid degradation of this GPCR. Unlike the situation in yeast cells, in HEK 293 cells α-factor stimulates internalization of Ste2, but does not lead to any significant reduction in the cellular complement of the receptor [4]. Yeast cells express no receptor tyrosine kinases and Ste2 has evolved in their absence. We expressed yeast α-factor pheromone receptors (Ste2) in human HEK293 cells in order to employ Ste2 as a target heptahelical receptors into which motifs found in mammalian GPCRs could be inserted in order to explore the contribution(s) of the inserted domain(s) to receptor trafficking by receptor tyrosine kinases.

## Results

**Figure 1:**
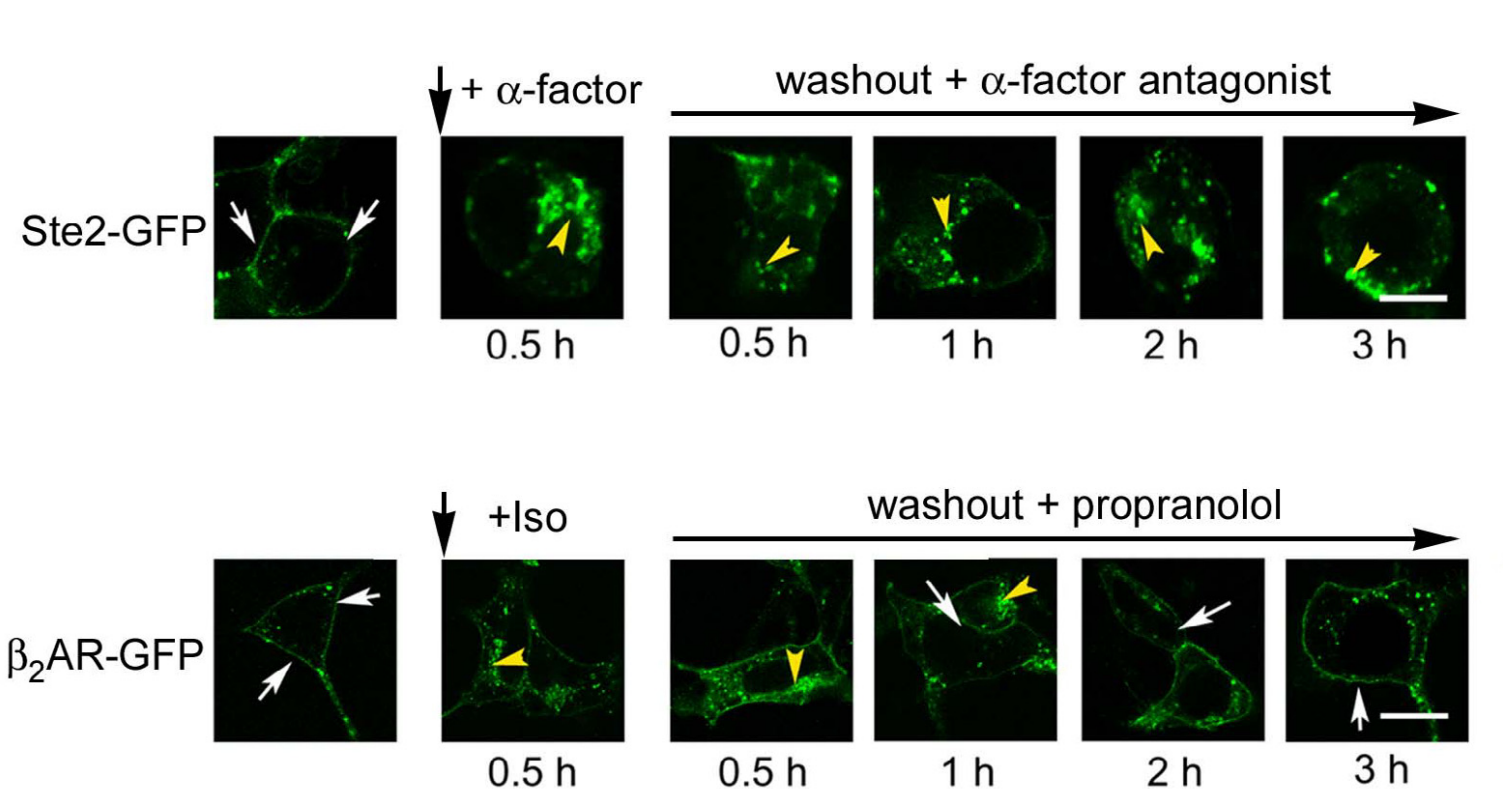
**Trafficking of yeast Ste2 in human HEK293 cells in response to α-factor: analysis by confocal microscopy**. HEK cells were transiently transfected to express either Ste2-GFP or β_2_AR-GFP. Unstimulated cells display receptors that are largely localized to the cell membrane (white arrows denote cell membrane-localized receptors). Cells expressing yeast Ste2-GFP and challenged with α-factor (10 μM, upper panel) as well as those expressing β_2_AR-GFP and challenged with beta-adrenergic agonist isoproterenol (Iso, 10 μM; lower panel) for 0.5 hr displayed frank internalization (yellow arrowheads denote internalized receptors). Agonist ligands were then washed from the media and the cells treated the appropriate antagonist ligand. Possible recycling of internalized receptors back to the cell membrane was followed for 3 hours. For cells expressing Ste2-GFP, the *a*-factor antagonist (des-Trp, Ala-3 analog of *a*-factor, 10 μM) was added after wash-out and the cells were monitored at 0.5 (panel *c*), 1 (panel *d*), 2 (panel *e*), and 3 (panel *f*) hour time periods after wash-out of agonist. For cells expressing β_2_AR-GFP, the β-adrenergic antagonist propranolol (10 μM) was added after wash-out of isoproterenol and the cells were monitored at 0.5 (panel *i*), 1 (panel *j*), 2 (panel *k*), and 3 (panel *l*) hour time periods post wash-out of agonist. The images displayed are from a single experiment, representative of more than five replicate, separate experiments. Bar equals 10 μm.

We tested if the internalized Ste2, not subject to rapid degradation, recycled from the cytosolic compartment back to the cell membrane once agonist action was terminated, a property of most members of the superfamily of mammalian GPCRs [5]. HEK293 cells were transiently transfected with either green fluorescent protein-tagged Ste2 (Ste2-GFP) or GFP-tagged β_2_AR (β_2_AR-GFP, figure 1) and viewed by confocal microscopy. Expression of each receptor was assayed by immunoblotting and radioligand binding (not shown). In the absence of α-factor, Ste2 were expressed largely on the cell membrane (figure 1; white arrows highlight cell surface-localized receptors, Ste2-GFP). Upon 30 min of stimulation with α-factor (figure 1; α-factor, 10 μM, 0.5 h) the bulk of Ste2 was observed to be internalized (figure 1; yellow arrowheads highlight internalized receptors). Cells were washed free of agonist and incubated with α-factor antagonist (des-Trp, Ala-3 α-factor mutant, 10 μM) and the localization of Ste2 monitored at 0.5, 1, 2, and 3 hours later (figure 1; washout + α-factor antagonist). Internalized Ste2 remains localized in the cytoplasmic compartment at each of these times following wash-out of agonist and addition of antagonist, *i.e*., no significant recycling of the internalized Ste2 back to the cell membrane was observed.

Like Ste2, β_2_AR-GFP expressed in HEK293 cells was largely confined to the cell membrane (figure 1; white arrows highlight cell surface-localized receptors). In the presence of β-adrenergic agonist (figure 1; isoproterenol, 10 μM, 0.5 h), β_2_AR displays well-known agonist-induced internalization (figure 1; yellow arrowheads highlight internalized receptors). Wash-out of β-adrenergic agonist and addition of a β-adrenergic antagonist (figure 1; wash-out + propranolol, 10 μM), in sharp contrast to the situation observed for Ste2, was followed by a rapid and nearly complete recycling of the β_2_AR to the cell membrane within 3 hours. For these studies, the medium was supplemented with the protein synthesis inhibitor cycloheximide (20 μg/ml) to suppress new synthesis of receptors. Thus, the ability to express yeast Ste2 in mammalian cells provides a novel template enabling study of receptor motifs necessary for recycling of mammalian GPCRs from the cytoplasmic compartment to the cell membrane.

**Figure 2:**
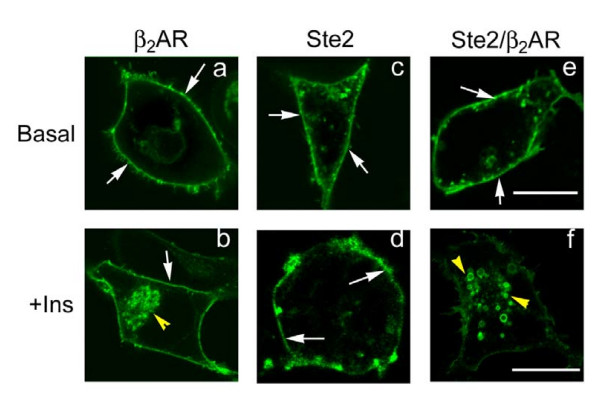
**Trafficking of yeast Ste2, human β_2_AR, and a yeast Ste2/human β _2_AR chimera expressed in human HEK293 cells in response stimulation by insulin: analysis by confocal microscopy**. HEK293 cells were transiently transfected to express either β_2_AR-GFP (panels *a, b*), Ste2-GFP (panels *c, d*), or the Ste2/β_2_AR-GFP chimeric receptor (panels *e,f*). For each of these GPCRs, localization of the receptors in the untreated cells was dominant at the cell membrane (white arrows). Cells were treated without (panels *a, c, e*) or with (panels *b, d, f*) insulin (100 nM) for 0.5 h and followed by confocal microscopy. Insulin stimulates internalization of the β_2_AR-GFP (panel *b*) and of the Ste2/β_2_AR-GFP chimeric receptor (panel *f*), but not of Ste2-GFP (panel *d*). The images displayed are from a single experiment, representative of more than five replicate, separate experiments. Bar equals 10 μm.

Mammalian cells express members of the receptor tyrosine kinases, including epidermeal growth factor receptor, insulin receptor, platelet-derived growth factor receptor, and others that have been shown to regulate GPCRs via tyrosine phosphorylation [6]. A prominent example of regulation of GPCRs by receptor tyrosine kinases is the well known insulin counterregulation of β_2_ARs [6]. When expressed in HEK293 cells, β_2_ARs display tyrosine phosphorylation and rapid internalization in response to insulin [6], as is observed in hamster *vas deferens *smooth muscle cells, human A431 cells, and other cell lines [7]. As shown by confocal microscopy (figure 2), β_2_AR-GFP is localized to the cell membrane (figure 2, panel *a*; "Basal", white arrows highlighting cell surface-localized receptors). The β_2_AR are internalized in response to insulin (100 nM, 0.5 h), although some cell surface-localized β_2_AR are still observed following insulin stimulation (figure 2; panel *b*, see white arrows). The ability of receptor tyrosine kinases that respond to insulin (shown), IGF-1 [8], and other growth factors to provoke the internalization of GPCRs, such as the β_2_AR, is referred to as "counterregulation" [7].

Yeast cells, in contrast, do not express receptor tyrosine kinases (*e.g.*, epidermal growth factor receptor, insulin receptor, and IGF-I receptor), making Ste2 an ideal target for the study of the protein motifs through which mammalian tyrosine kinases regulate GPCRs. The Ste2-GFP is localized to the cell membrane in unstimulated conditions (figure 2; panel *c*), but unlike the β_2_AR does not show internalization in response to insulin treatment (figure 2; panel *d*). The observed inability of insulin to regulate trafficking of Ste2 confirmed our base premise about this yeast receptor and also created the opportunity to employ the Ste2 GPCR as a model in which to probe the protein motifs by which insulin, as well as other growth factors, can regulate the internalization and recycling of GPCR substrates.

A chimeric receptor that makes use of the Ste2 exofacial and transmembrane segments with substitution of the cytoplasmic domains of Ste2 with those of the β_2_AR was engineered and we tested if the Ste2/β_2_AR chimera would enable insulin-stimulated internalization. The Ste2/β_2_AR chimera, as presumed, lost its ability to transduce α-factor stimulation into activation of the mitogen-activated protein kinases Erk1,2 (data not shown). The GFP-tagged Ste2/β_2_AR chimera, studied by confocal microscopy, was largely localized to the cell membrane (figure 2; panel *e*) in the absence of added hormones. In response to stimulation by insulin (100 nM) for 30 min, the β_2_AR displayed marked internalization (figure 2; panel *b*); Ste2 did not display any significant insulin-stimulated internalization (figure 2; panel *d*). The Ste2/β_2_AR chimera, in contrast, displayed prominent internalization in response to insulin stimulation (figure 2; panel *f*). Evolving in the absence of mammalian protein kinases and adaptor proteins operating in GPCR receptor trafficking [9], Ste2 provides a useful target for molecular analysis of protein motifs that act as substrates for tyrosine kinases and adaptor molecules.

## Discussion

Ste2 was found to display agonist-induced internalization in response to stimulation of the cells with α-factor, but unlike the β_2_-adrenergic receptors (β_2_AR) and most other GPCRs, failed to demonstrate recycling to the cell membrane following removal of its agonist, providing a novel system for investigation of the motifs critical for GPCR recycling. Insulin stimulates, via activation of its receptor tyrosine kinase and phosphorylation of its substrate receptors [10], a counterregulation of GPCRs, including the β_2_AR [11]. Yeast Ste2 expressed in animal cells, in contrast, does not display insulin-stimulated internalization, having evolved in the absence of receptor tyrosine kinases of the nature of the insulin, EGF, IGF-I, and PDGF receptors. A chimeric receptor composed of the exofacial and transmembrane segments of Ste2 onto which the cytoplasmic domains of β_2_AR are substituted was engineered and successfully expressed in animal cells. Unlike Ste2, the chimeric receptor was found to display insulin-stimulated internalization. These results provide a proof-of-concept for the utility of yeast Ste2 expressed in animal cells as a tool for study of mammalian protein kinases and adaptors involved in GPCR trafficking.

## Conclusion

The yeast pheromone Ste2 GPCR provides a useful example of primitive receptor regulation, in the sense that the receptor biology is almost linear from agonist binding to receptor degradation, *i.e*., binding > activation > internalization > ubiquitination > degradation by the proteosome. In higher organisms, additional new layers of regulation can be observed in which retention of the GPCR and/or its recycling from internalization are essential to maintaining proper signaling. Obviously, for neurotransmitter receptors at the synapse, single-hit biology, in which the receptor is activated and shortly thereafter degraded, would pose an enormous problem for biosynthetic capacity of the neuron. Resensitization and recycling of internalized GPCRs is more the norm for the mammalian systems and the central question arises, how do mammalian cells regulate cell-surface retention times and recycling capabilities? In the current work, we made use of the knowledge that yeast GPCRs operate in the absence of input from receptor tyrosine kinases that are well known to play essential roles in modulating GPCRs in higher organisms. The yeast Ste2 provided a "clean" slate, since once successfully expressed in animal cells, it demonstrated functional downstream signaling and agonist-induced internalization. But unlike the situation in yeast cells, Ste2 internalization provoked neither rapid destruction of this GPCR nor any obvious recycling of the internalized receptor back to the cell membrane for reutilization. Since regulation of GPCR trafficking by receptor tyrosine kinases (*e.g.*, insulin receptor) can involve direct tyrosine phosphorylation of the GPCR as well as phosphorylation of the GPCR by downstream serine/threonine protein kinases (*e.g*., protein kinase B/Akt), understanding the full range of protein motifs, phosphorylations, and downstream interactions with adaptor proteins (*e.g.*, Grb2) may be best revealed by introducing regions of targeted GPCRs into Ste2 and probing the regulation of the chimera in the animal cells. We succeeded in conferring counterregulation by insulin to yeast Ste2 by creating a chimera with cytoplasmic domains substituted by the β_2_-adrenergic receptor. The protein kinases, phosphatases, and adaptor molecules necessary for resensitization/recycling of mammalian GPCRs can be screened by study of such internalized Ste2 chimera. We provide the proof-of-concept for this strategy and suggest that Ste2 chimera may prove invaluable in many such efforts to probe the structural determinants involved in affecting GPCR cell surface retention times and GPCR recycling, essential aspect of GPCR biology in higher organisms.

## Methods

### Plasmids

The STE2 gene was amplified by PCR by using plasmid pDB02 [12] as the template and a pair of primers designed with *Nhe*I or *Bam*HI linkers. The PCR product was digested with *Nhe*I and *BamH*I and cloned into the unique *Nhe*I/*Bam*HI sites of peGFP-N1 expression vector (Clontech). The plasmid encoding the enhanced GFP-tagged human β_2_AR (in pCDNA3) has been fully characterized in a variety of mammalian cell lines [13,14]. A chimeric receptor (Ste2/β_2_AR) was generated by replacement of the three cytoplasmic loops and the C-terminus of the Ste2 with the corresponding regions of the β_2_AR using overlapping extension PCR and cloned into the *Nhe*I/*Bam*HI sites of the pEGFP-N1. The sequence of each construct was confirmed by DNA sequencing.

### Cell culture and transfections

The human embryonic kidney HEK293 cells were maintained in Dulbecco's Modified Eagles Medium (DMEM) supplemented with 10% fetal bovine serum (FBS, HyClone), penicillin (60 μg/mL) plus streptomycin (100 μg/mL), and grown in a humidified atmosphere of 5% CO_2 _and 95% air at 37°C. For transfections, cells were seeded at a density of 2 × 10^6 ^cells/100-mm dish, cultured for 24 h, and transiently transfected using LipofectAMINE (Invitrogen) according to the manufacturer's recommendations. The cells then were cultured in the growth medium for 48 h.

### Confocal microscopy

For the confocal microscopy studies, cells expressing GFP-tagged receptors were grown on Nunc^® ^chambered coverglasses. Following an overnight serum starvation, cells were treated as indicated and fixed with ice-cold methanol for 2 min at -20°C. Images were taken with Zeiss 510 inverted confocal microscope (60×, oil immersion). Images were imported as tiff.files, processed and prepared in Adobe Photoshop^© ^5.5.

## Competing interests

The authors declare that they have no competing interests.

## Authors' contributions

DY engineered the receptor expression constructs and drafted the original manuscript. ES provided direction about the conditions for expression of receptors and expertise in the confocal microscopy. HYW helped to conceive the study and participated in the design of the experiments. CCM helped to conceive the study, developed and edited the manuscript. All authors read and approved of the final manuscript.

## Abbreviations

β_2_AR, beta_2_-adrenergic receptors; GFP, enhanced green fluorescent protein; GPCR, G protein-coupled receptors; HEK, human embryonic kidney.
